# Acknowledgement of manuscript reviewers 2019

**DOI:** 10.18332/tid/118190

**Published:** 2020-02-14

**Authors:** Israel Agaku, James Elliott Scott

**Affiliations:** 1 Harvard School of Dental Medicine, Boston, United States; 2 University of Manitoba, Winnipeg, Canada; 3 Manitoba Institute of Child Health, Winnipeg, Canada

## CONTRIBUTING REVIEWERS

The editors of Tobacco Induced Diseases would like to thank all our reviewers who have contributed to the journal in Volume 17 (2019).

**Abdullah, Abeer**

United States

**Abu-Helalah, Munir**

Jordan

**Agarwalla, Rashmi**

India

**Agnihotri, Rupali**

India

**Akers, Laura**

United States

**Akram, Zohaib**

Pakistan

**Alamer, Nora**

United States

**Aldosari, Muath**

United States

**Aldukhail, Shaikha**

United States

**Alexander, Adam**

United States

**Aleyan, Sarah**

Canada

**Alhazmi, Hesham**

United States

**Allem, Jon-Patrick**

United States

**Alotaibi, Saad**

United States

**Amarasinghe, Hemantha**

Sri Lanka

**Anand, Vivek**

United States

**Anderson, Chastain**

United States

**Arrazola, René**

United States

**Aryal, Umesh**

Nepal

**Ashwin, Cathy**

United Kingdom

**Aune, Dagfinn**

United Kingdom

**Azab, Mohammad**

Jordan

**Bahdila, Dania**

United States

**Bahrami, Golnosh**

Denmark

**Balappanavar, Aswini**

India

**Balderrama, Fanor**

Canada

**Balwicki, Łukasz**

Poland

**Bayer, Oliver**

Germany

**Beklen, Arzu**

Turkey

**Bello, Dhimiter**

United States

**Bennett, Brooke**

United States

**Berglund, Kristina**

Sweden

**Bhaskar, Ravi Kumar**

Nepal

**Bhisey, Rajani**

India

**Bizoń, Anna**

Poland

**Bledsoe, Linda**

United States

**Borracci, Raúl**

Argentina

**Bostan, Pinar**

Turkey

**Bowker, Katharine**

United Kingdom

**Briganti, Mike**

United States

**Brown, Elizabeth**

United States

**Brown, Jennifer**

United States

**Buduneli, Nurcan**

Turkey

**Callahan-Lyon, Priscilla**

United States

**Campbell, Kasia**

United Kingdom

**Campo, Laura**

Italy

**Cantrell, Jennifer**

United States

**Carracedo, Patricia**

Spain

**Chean, Kooi-Yau**

Malaysia

**Chen, Julia**

United States

**Chen, Mei-Ling**

Taiwan

**Cheng, Li**

China

**Cheung, Yee Tak Derek**

Hong Kong

**Chowdhury, Parimal**

United States

**Coleman, Sulamunn**

United States

**Conner, Mark**

United Kingdom

**Cruvine, Erica**

Brazil

**Cucullo, Luca**

United States

**Dautzenberg, Bertrand**

France

**De Gennaro, Luigi**

Italy

**Desai, Masoom**

United States

**Dewhirst, Timothy**

Canada

**Dewi, Susanna**

Indonesia

**Donahoe, J. Travis**

United States

**Dorotheo, Edgardo Ulysses**

Philippines

**Drehmer, Jeremy**

United States

**Driezen, Pete**

Canada

**Drovandi, Aaron**

Australia

**Ebersole, Jeffrey**

United States

**Edwards, Sarah**

Canada

**Eldridge, Gloria**

United States

**Erdemir, Ebru**

Turkey

**Escario, José**

Spain

**Ferron, Joelle C.**

United States

**Fetterman, Jessica**

United States

**Filippidis, Filippos**

United Kingdom

**Fischer, Florian**

Germany

**Francis, Diane**

United States

**Fritz, Deborah**

United States

**Fujita, Masaki**

Japan

**Gallus, Silvano**

Italy

**Ganz, Ollie**

United States

**Gathecha, Gladwell**

Kenya

**Gichuki, Judy**

Kenya

**Gilbert, Hazel**

United Kingdom

**Girvalaki, Charis**

Greece

**Goodall, Catherine**

United States

**Gordon, Kobie**

United States

**Gorini, Giuseppe**

Italy

**Gregory, Richard**

United States

**Hanioka, Takashi**

Japan

**Hanke, Wojciech**

Poland

**Hasegawa, Koji**

Japan

**Hedman, Linnea**

Sweden

**Hegazi, Fahad**

United States

**Heshmat Ghahderijani, Bahareh**

Iraq

**Heydari, Gholamreza**

Iran

**Hiscock, Rosemary**

United Kingdom

**Hitchman, Sara**

United Kingdom

**Holliday, Richard**

United Kingdom

**Hsu, P.T.**

Taiwan

**Huang, Tianyi**

United States

**Hummel, Karin**

Netherlands

**Isiozor, Nzechukwu**

Finland

**Javed, Fawad**

United States

**Jongenelis, Michelle**

Australia

**Kaai, Susan**

Canada

**Kabir, Zubair**

Ireland

**Kachhwaha, Khushboo**

India

**Kaito, Chikara**

Japan

**Karam-Hage, Maher**

United States

**Karelitz, Joshua**

United States

**Kathuria, Hasmeena**

United States

**Katsaounou, Paraskevi**

Greece

**Kazi, Tasneem G.**

Pakistan

**Keegan, Conor**

Ireland

**Kennedy, Erinne**

United States

**Khan, Amer**

Malaysia

**Khazdair, Mohammad**

Iran

**King, Bill**

Australia

**Kobeissy, Firas**

United States

**Korona-Glowniak, Izabela**

Poland

**Koziel, Slawomir**

Poland

**Krishnan, Nandita**

United States

**Kristina, Susi**

Indonesia

**La Torre, Giuseppe**

Italy

**Laestadius, Linnea**

United States

**Lam, Tai**

China

**Lee, Chung-won**

United States

**Lee, Hyun Sook**

Republic of Korea

**Lee, Joseph**

United States

**Lee, Kelley**

Canada

**Lee, Sungkyu**

Republic of Korea

**Leite, Fabio**

Denmark

**Lewis, Jane**

United States

**Leys, Didier**

France

**Li, Meng**

Japan

**Li, Qiang**

China

**Liang, Lan**

United States

**Lim, Kuang Hock**

Malaysia

**Liu, Jian-Ping**

China

**Lopez, Maria J.**

Spain

**López Nicolás , Ángel**

Spain

**Luk, Tzu Tsun**

Hong Kong

**Lum, Alistair**

Australia

**Mackay, Judith**

China

**Maddox, Raglan**

Australia

**Malouff, John**

Australia

**Mannino, David**

United States

**Mantey, Dale**

United States

**Martinez-Vispo, Carmela**

Spain

**Masjedi, Mohammad**

Iran

**Mayer, Otto**

Czech Republic

**Mayhew, Kathryn**

United States

**Mayyas, Fadia**

Jordan

**McCarthy, William**

United States

**McDaniel, Patricia**

United States

**McGinnis, Kathleen**

United States

**McKinstry, Kai**

United States

**Mdege, Noreen**

United Kingdom

**Mennis, Jeremy**

United States

**Miller, Caroline**

Australia

**Mitrou, Francis**

Australia

**Mohammed, Alqahtani**

United Arab Emirates

**Mohammed, Mohammed**

Saudi Arabia

**Mohan, Surapaneni**

India

**Monson, Eva**

Canada

**Moshammer, Hanns**

Austria

**Nakkash, Rima T.**

Lebanon

**Nascimento, Gustavo**

Denmark

**Nelson, Jevae**

United States

**Ngan, Tran**

Vietnam

**Northrup, Thomas**

United States

**Nuyts, Paulien**

Netherlands

**Obidike, Chikaodili**

United States

**Odani, Satomi**

United States

**Oliveira Serra, Maria**

Brazil

**Oliver, Jason**

United States

**Olowookere, Samuel**

Nigeria

**Otoukesh, Babak**

Germany

**Pandey, Arti**

Nepal

**Papadakis, Sophia**

Canada

**Patelarou, Evridiki**

United Kingdom

**Pénzes, Melinda**

Hungary

**Pergadia, Michele**

United States

**Phetphum, Chakkraphan**

Thailand

**Pinilla Domínguez, Jaime**

Spain

**Pitayarangsarit, Siriwan**

Thailand

**Pitha, Jan**

Czech Republic

**Plopper, Charles**

United States

**Pollok, Richard**

United Kingdom

**Popova, Lucy**

United States

**Prabandari, Yayi**

Indonesia

**Protano, Carmela**

United States

**Purves, Richard**

United Kingdom

**Raina, Romshi**

India

**Ranabhat, Chhabi**

Nepal

**Rasmussen, Mette**

Denmark

**Ravara, Sofia**

Portugal

**Rayens, Mary**

United States

**Readshaw, Anne**

United Kingdom

**Reutter, Mirjam**

Germany

**Reynolds, Ciara**

Ireland

**Rezaei, Satar**

Iran

**Riehm, Kira**

United States

**Rosa, Rodrigo**

Brazil

**Ruhil, Rohini**

India

**Salam, Hira**

Pakistan

**Salazar, Juan**

Venezuela

**Salepci, Banu**

Turkey

**Salous, Moaid**

United States

**Sangthong, Rassamee**

Thailand

**Santulli, Gaetano**

United States

**Satomura, Kazunari**

Japan

**Scott, David**

United States

**Scott, James Elliot**

Canada

**Sharma, Shreya**

India

**Sirichotiratana, Nithat**

Thailand

**Stimson, Gerry**

United Kingdom

**Stoklosa, Michal**

United States

**Strutt, Tara**

United States

**Sun, Shaojing**

China

**Surani, Nikita**

India

**Suryadhi, Made**

Japan

**Susumu, Sato**

Japan

**Suzuki, Kohta**

Japan

**Sæbø, Gunnar**

Norway

**Takeda, Flavio**

Brazil

**Tatullo, Marco**

Italy

**Tete, Giulia**

Italy

**Thomson, Neil**

United Kingdom

**Tonnesen, Philip**

Denmark

**Tonstad, Serena**

Norway

**Trofor, Antigona**

Romania

**Tsai, Ching-Hui**

Taiwan

**Ucar, Elif**

Turkey

**Ulbricht, Sabina**

Germany

**Valente, Nicola**

United States

**Valiente, Roberto**

Spain

**van Dessel, Pieter**

Belgium

**Vazquez-Nava, Francisco**

Mexico

**Vitória, Paulo**

Portugal

**Vohra, Fahim**

Saudi Arabia

**Vouros, Ioannis**

Greece

**Wang, Bin**

China

**Wang, Jian-Bing**

China

**Wang, Li**

China

**Warnakulasuriya, Saman**

United Kingdom

**Whitehead, Todd**

United States

**Wiernik, Emmanuel**

France

**Wu, Ping**

Canada

**Wu, Yongda**

Hong Kong

**Xiao, Hong**

United States

**Xiao, Lin**

China

**Xu, Xiaoxin**

China

**Yadav, Amit**

India

**Yanagita, Manabu**

Japan

**Yaprak, Emre**

Turkey

**Yoshimi, Itsuro**

Japan

**Yuqing, Zheng**

United States

**Zacharasiewicz, Angela**

Austria

**Zatonski, Witold**

Poland

**Zeebari, Zangin**

Sweden

**Zhang, Yuwei**

United States

**Zheng, Pinpin**

China

**Zhou, Suzanne**

Australia

**Zou, Chunbin**

United States

